# A Normalization Framework for Emotional Attention

**DOI:** 10.1371/journal.pbio.1002578

**Published:** 2016-11-21

**Authors:** Xilin Zhang, Shruti Japee, Zaid Safiullah, Nicole Mlynaryk, Leslie G. Ungerleider

**Affiliations:** Laboratory of Brain and Cognition, National Institute of Mental Health, National Institutes of Health, Bethesda, Maryland, United States of America; Vanderbilt University, UNITED STATES

## Abstract

The normalization model of attention proposes that attention can affect performance by response- or contrast-gain changes, depending on the size of the stimulus and attention field. Here, we manipulated the attention field by emotional valence, negative faces versus positive faces, while holding stimulus size constant in a spatial cueing task. We observed changes in the cueing effect consonant with changes in response gain for negative faces and contrast gain for positive faces. Neuroimaging experiments confirmed that subjects’ attention fields were narrowed for negative faces and broadened for positive faces. Importantly, across subjects, the self-reported emotional strength of negative faces and positive faces correlated, respectively, both with response- and contrast-gain changes and with primary visual cortex (V1) narrowed and broadened attention fields. Effective connectivity analysis showed that the emotional valence-dependent attention field was closely associated with feedback from the dorsolateral prefrontal cortex (DLPFC) to V1. These findings indicate a crucial involvement of DLPFC in the normalization processes of emotional attention.

## Abstract 2

Using a combination of psychophysics and functional MRI, this study reveals that emotional attention interacts with normalization processes depending on emotional valence (positive or negative faces), best explained by feedback modulation from the dorsolateral prefrontal cortex.

## Author Summary

Attentional selection is the mechanism by which the subset of incoming information is preferentially processed at the expense of distractors. The normalization model of attention suggests that attention-triggered modulatory effects on sensory responses in the visual cortex depend on two factors: the stimulus size and the attention field size. However, little is known regarding whether emotional attention shapes perception by means of the normalization framework. To test this hypothesis, we manipulated the attention field by emotional valence—negative faces versus positive faces—while holding the stimulus size constant in a spatial cueing task. We observed that attention increased response gain for negative faces, with the largest cueing effects occurring at high contrasts and little to no effect at low and mid-contrasts; however, attention increased contrast gain for positive faces, with the largest cueing effects occurring at mid-contrasts and little to no effect at low and high contrasts. A complementary neuroimaging experiment confirmed that subjects' attention fields were narrowed for negative faces and broadened for positive faces. Across subjects, the self-reported emotional strength of negative faces and positive faces correlated, respectively, both with response-gain and contrast-gain changes and with narrowed and broadened attention fields in the primary visual cortex. Mechanistically, we found that the emotional valence-dependent attention field was closely associated with feedback from the dorsolateral prefrontal cortex to the primary visual cortex. Our findings provide evidence for a normalization framework for emotional attention and for the critical role of feedback from the prefrontal cortex to the early visual cortex in this normalization.

## Introduction

Attentional selection is the mechanism by which the subset of incoming information is preferentially processed at the expense of distractors. Numerous studies have suggested that attentional selection modulates both visual performance and neuronal activity in striate and extrastriate visual cortices [1]. However, studies have found disparate attentional selection effects on stimulus-evoked neural responses, such as the contrast-response function (CRF) [2,3]. Some have reported that attentional selection primarily enhances neural responses to high-contrast stimuli (response gain) [4–9], whereas others have reported that attentional selection primarily enhances neural responses to medium-contrast stimuli (contrast gain) [2,3,10,11]. Still others have reported that attentional selection either enhances the entire contrast range or produces a combination of both response-gain and contrast-gain changes [12–16].

The normalization model of attention suggests that these seemingly conflicting modulatory effects of attention on sensory responses in the visual cortex may depend on two factors: the stimulus size and the attention field size [6,17–19]. Changes in the relative size of these two factors can tip the balance between neuronal excitatory and inhibitory processes, thereby resulting in response-gain changes, contrast-gain changes, or various combinations of the two [19]. Specifically, this model predicts that attention increases response gain when the stimulus is large and the attention field is small and increases contrast gain when the stimulus is small and the attention field is large. Previous psychophysical [17] and electroencephalography [20] studies have reported that the pattern of both behavioral performance and steady-state visual evoked potentials is consistent with the normalization model of attention. However, little is known about whether emotional attention also shapes perception by means of the normalization framework.

Emotional stimuli, both negative and positive emotion, tend to attract attention in humans as well as other primates [21–26]. However, there is a critical distinction between the perceptual correlates of negative and positive emotions, with negative emotion narrowing and positive emotion broadening the scope of attention or perception [27–30]. For example, negative emotion shows lower and positive emotion shows higher sensory responses to unattended extrafoveal stimuli than neutral emotion [31]. The narrowing of the attention field by negative emotion is sometimes referred to as “weapon focus,” in which peripheral details of stimuli are more poorly encoded, as measured in later memory [32] and repeated adaptation [31]. Similarly, negative emotion is associated with a greater tendency to perceive local components of visuospatial stimuli [33], whereas positive emotion is associated with a greater tendency to perceive their global components [34]. Therefore, emotional stimuli, negative versus positive, offer a unique opportunity to change the size of the attention field relative to the stimulus, differentially modulating the gain of attentional selection.

Here, the size of the attention field was manipulated by emotional valence—negative faces versus positive faces—while the stimulus size was held constant, and the stimulus contrast was varied in a spatial cueing task [19,35]. We measured the gain pattern of CRFs on the spatial cueing effect derived by the emotional faces and empirically revealed an interaction between emotion and attention: gain modulation depended on emotional valence, with a change in the spatial cueing effect consonant with a change in response gain for negative faces and a change in contrast gain for positive faces. A functional magnetic resonance imaging (fMRI) experiment confirmed that subjects’ attention fields were narrowed and broadened by negative faces and positive faces, respectively, as indexed by the decreased and increased primary visual cortex (V1) responses to flanking gratings. Furthermore, the self-reported emotional strength of the emotional faces significantly correlated with the psychophysical gain modulations, and with the V1 blood oxygenation-level-dependent (BOLD) signal changes, across individual subjects. Finally, effective connectivity analysis showed that emotional valence controlled the attention field through the modulation of feedback from the dorsolateral prefrontal cortex (DLPFC) to V1. These findings indicate that emotional attention interacts with the normalization processes depending on emotional valence, which is best explained by feedback modulation to the visual cortex from DLPFC.

## Results

### Psychophysical Experiments

**Fig 1:**
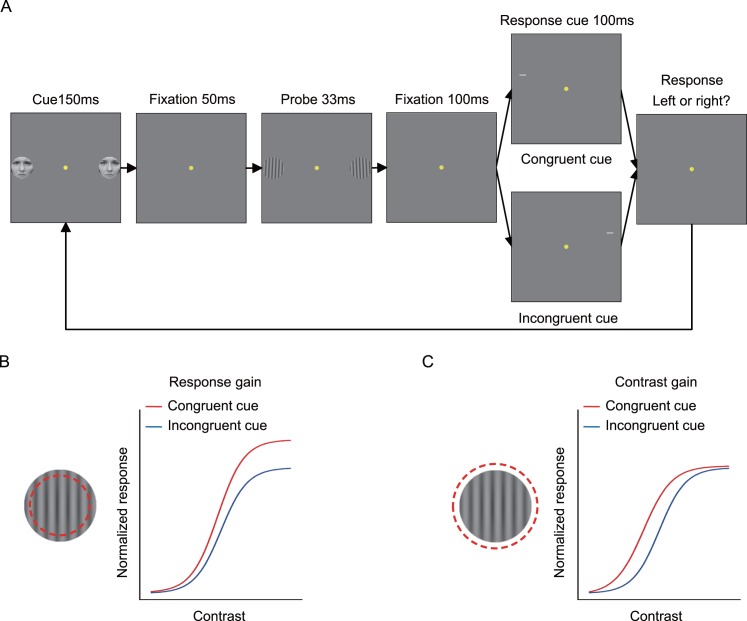
Psychophysical protocol and the normalization model of attention modulated by emotional valence. (A) Psychophysical protocol. A pair of faces (one emotional and the other neutral; the faces of a coauthor here are for illustration purposes only; they were not used in the experiments. The experimental faces were chosen from the NimStim Set of Facial Expressions: http://www.macbrain.org/resources.htm [36] and could not be published under the Creative Commons Attribution license) were presented for 150 ms, followed by a 50 ms fixation interval. Then, a pair of gratings were presented for 33 ms in the left and right hemifields, one of which was the target. Subjects were asked to discriminate the orientation of the target grating and received auditory feedback if their response was incorrect. The target location was indicated by a peripheral 100 ms response cue (0.5° white line) above one of the grating locations, but not at the grating location to avoid masking. A congruent cue was defined as a match between the emotional face location and response cue location; an incongruent cue was a mismatch. (B) Relative to the stimulus size, the attention field was narrowed by negative faces. Under this configuration, the normalization model predicts a response-gain shift, with the largest effects occurring at high contrasts and little to no effect at low and mid-contrasts. (C) Relative to the stimulus size, the attention field was broadened by positive faces. Under this configuration, the normalization model predicts a contrast-gain shift, with the largest effects occurring at mid-contrasts and little to no effect at low and high contrasts. The dashed red circles indicate simulated attention field size.

In the psychophysical experiment, subjects performed an orientation discrimination task on one of two target grating patches; each was presented at five different contrasts (the contrasts of both gratings were identical on any given trial and covaried across trials in random order). Covert attention (without eye movements, S1 Fig) was captured by the emotional face (negative or positive), which also modulated the attention field: negative faces narrowed and positive faces broadened the attention field (Fig 1B and 1C). A response cue at the stimulus offset indicated the target location, yielding congruent cue (the emotional face matched the response cue) and incongruent cue (mismatched) conditions (Fig 1A). Comparing performance accuracy (*d′*) for congruent and incongruent trials revealed the spatial cueing effect for each target contrast.

**Fig 2:**
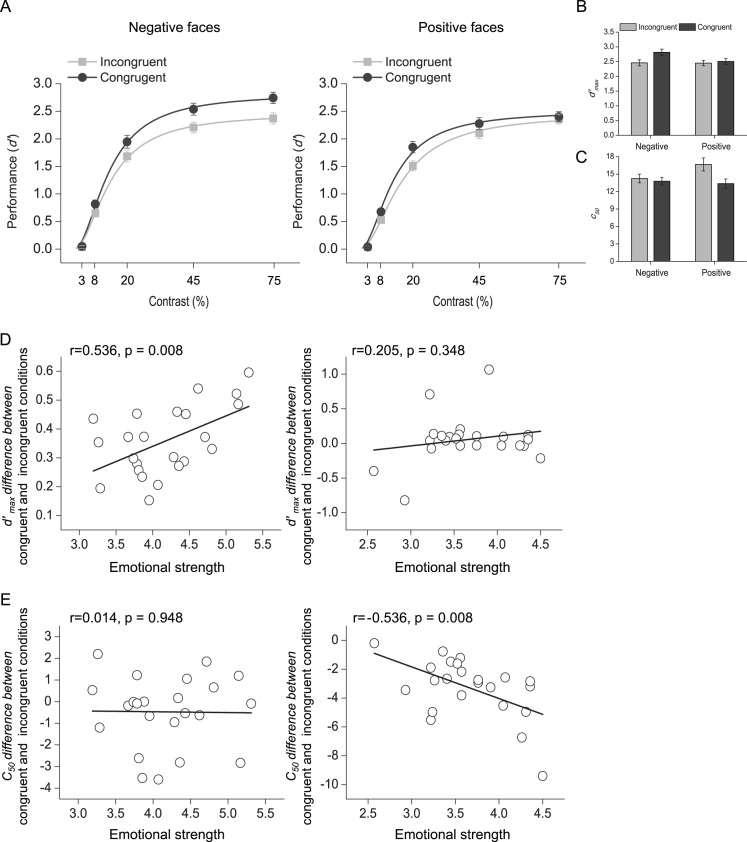
Spatial cueing effects on performance (*d'*) as a function of contrast. (A) Mean *d'* plotted as psychometric functions of stimulus contrast and emotional valence (negative, left; positive, right) for congruent and incongruent trials. *d'*_max_: asymptotic performance at high-contrast levels; *c*_50_: the contrast yielding half-maximum performance. (B) *d'*_max_ and (C) *c*_50_ for trial conditions and emotional valence. Error bars denote 1 standard error of the mean (SEM) calculated across subjects. (D) Correlations between the mean self-reported emotional strength of the faces and the *d'*_max_ differences between congruent and incongruent trials across individual subjects. (E) Correlations between the mean self-reported emotional strength of the faces and the *c*_50_ differences between congruent and incongruent trials across individual subjects. The data are in the Supporting Information (see S1 Data).

The mean *d′* plotted as psychometric functions of stimulus contrast and emotional valence are shown in Fig 2A: the negative emotion yielded a pattern that qualitatively resembled response gain (left), and the positive emotion yielded a pattern that qualitatively resembled contrast gain (right). The measured psychometric function for each emotional valence (negative and positive) and each trial condition (congruent and incongruent) was fit with the standard Naka–Rushton equation [37]. The two parameters *d'*_max_ (asymptotic performance at high-contrast levels) and *c*_50_ (the contrast yielding half-maximum performance) determined response gain and contrast gain, respectively. The exponent *n* (slope) was fixed at 2 in the current analysis [17,38]. The *d'*_max_ for emotional valence (negative and positive) and trial conditions (congruent and incongruent) are shown in Fig 2B and were submitted to a repeated-measures ANOVA with emotional valence and trial condition as within-subjects factors. The main effect of emotional valence (F_1, 22_ = 1.734, *p* = 0.201) was not significant, but the main effect of the trial condition (F_1, 22_ = 34.971, *p* < 0.001) and the interaction between these two factors (F_1, 22_ = 13.742, *p* = 0.001) were both significant. Further *t* tests showed that the *d'*_max_ of congruent trials was higher than that of incongruent trials (t_22_ = 14.422, *p* < 0.001) for negative emotion, but not for positive emotion (t_22_ = 0.789, *p* = 0.438); the *d'*_max_ for negative emotion was higher than that for positive emotion in the congruent trials (t_22_ = 2.181, *p* = 0.040), but not in the incongruent trials (t_22_ = 0.083, *p* = 0.934). Similarly, for the *c*_50_ (Fig 2C), the main effect of emotional valence was not significant (F_1, 22_ = 1.072, *p* = 0.312), but the main effect of the trial condition (F_1, 22_ = 40.884, *p* < 0.001) and the interaction between these two factors (F_1, 22_ = 30.950, *p* < 0.001) were both significant. Further *t* tests showed that the *c*_50_ of congruent trials was lower than that of incongruent trials for positive emotion (t_22_ = −7.676, *p* < 0.001), but not for negative emotion (t_22_ = −1.377, *p* = 0.182); the *c*_50_ for negative emotion was lower than that for positive emotion in the incongruent trials (t_22_ = −2.172, *p* = 0.041), but not in the congruent trials (t_22_ = 0.464, *p* = 0.647). These results thus suggest that gain modulation of attentional selection depends on emotional valence.

To evaluate further the role of emotional valence in the gain modulation of attention, we calculated the correlation coefficients between the self-reported emotional strength of the faces and psychophysical measures (*d'*_max_ and *c*_50_) across individual subjects. The self-reported emotional strength of negative faces significantly correlated with the *d'*_max_ difference between congruent and incongruent trials (r = 0.536, *p* = 0.008, Fig 2D, left), but not with the *c*_50_ difference between congruent and incongruent trials (r = 0.014, *p* = 0.948, Fig 2E, left). Conversely, the self-reported emotional strength of positive faces significantly correlated with the *c*_50_ difference between congruent and incongruent trials (r = −0.536, *p* = 0.008, Fig 2E, right), but not with the *d'*_max_ difference between congruent and incongruent trials (r = 0.205, *p* = 0.348, Fig 2D, right). These results thus demonstrate a close relationship between emotional valence and gain modulation of attentional selection (response-gain and contrast-gain changes in psychophysical performance). Furthermore, given that subjects performed the negative and positive sessions on two different days (the order of the two sessions was counterbalanced across subjects), we performed an additional analysis to confirm that the order of these two sessions did not influence our psychophysical results (S2 Fig).

### fMRI Experiments

**Fig 3:**
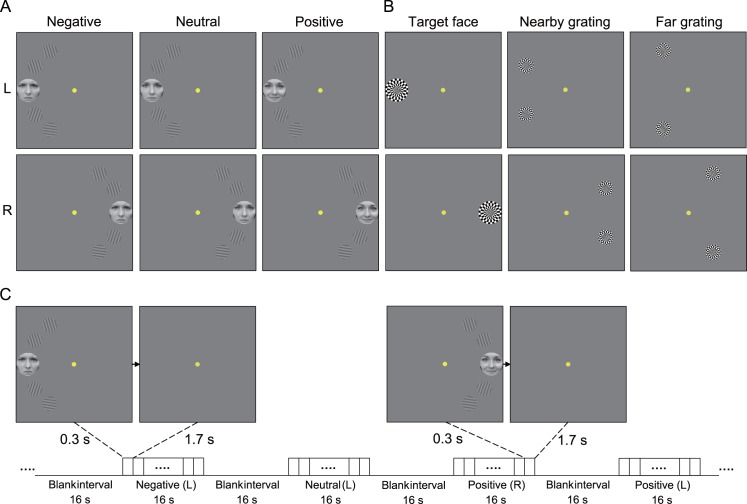
FMRI stimuli and protocol. (A) The target face (the faces of a coauthor here are for illustration purposes only; they were not used in the experiments) was centered in either the left or right visual field and was flanked by four gratings, two in the upper visual field and two in the lower visual field (L: left visual field; R: right visual field). (B) Region-of-interest (ROI) definition. The flickering patches were used to define ROIs in V1, corresponding to the target face (left), nearby gratings (middle), and far gratings (right). (C) FMRI procedure. Each face flanked by four gratings was presented for 0.3 s, followed by a 1.7-s fixation interval. Subjects were asked to discriminate the gender of each face (male or female) while maintaining central fixation throughout the trial.

To directly investigate whether negative emotion narrowed and positive emotion broadened subjects’ attention fields, a block-design fMRI experiment was designed to measure the V1 responses to task-irrelevant gratings (Fig 3A). Each run consisted of 12 stimulus blocks of 16 s, interleaved with 12 blank intervals of 16 s. There were 6 kinds of stimulus blocks: 2 (visual field: left/right) × 3 (emotional valence: negative/neutral/positive), and each stimulus block was randomly repeated two times in each run. For each type of emotional valence, data from the left and right visual fields were pooled together for analysis. Each stimulus block consisted of 8 trials; on each trial, a target face was centered at 4.65° eccentricity in the left or right hemifield and flanked by four gratings. The center-to-center distance between the target face and nearby gratings and between the target face and far gratings was 2.54° and 4.52°, respectively (Fig 3A and 3B). The target face and flanking gratings were presented for 0.3 s, followed by a 1.7-s fixation interval, and subjects were asked to discriminate the gender of the target face (male or female) while maintaining central fixation throughout the trial (Fig 3C). The accuracy rates (mean percent correct ± standard error of the mean [SEM]) were 91.35% ± 1.09%, 91.95% ± 0.86%, and 92.44% ± 1.15%, while the reaction times (mean reaction time ± SEM) were 813.06 ± 19.95 ms, 821.96 ± 19.09 ms, and 827.94 ± 20.16 ms for negative, neutral, and positive conditions, respectively. For these measurements, there was no significant difference (all *p* > 0.05) in subject performance among the three types of emotional valence of the target faces.

**Fig 4:**
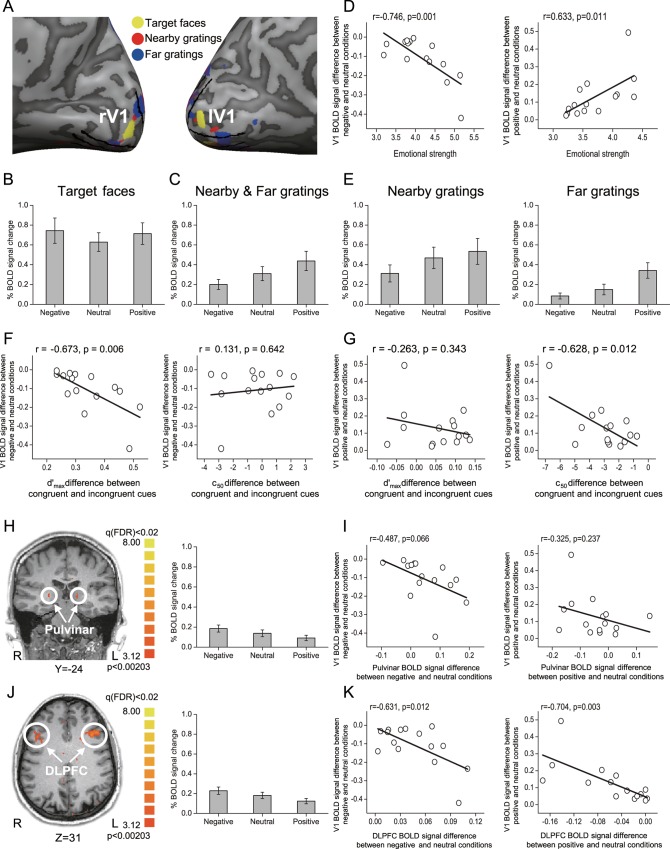
FMRI results. (A) ROIs. Flickering round-checkered patches with a full contrast were used to define the ROIs. They occupied the same spatial extents as the target face and the four flanking gratings. Cortical activations by the five patches are depicted in a representative inflated brain. The yellow, red, and blue areas correspond to ROIs of the target face, nearby gratings, and far gratings, respectively. The boundaries of V1, defined by retinotopic mapping, are indicated by the black lines. (B) BOLD signal amplitudes in V1 evoked by target faces. (C) BOLD signal amplitudes in V1 evoked by nearby and far gratings. (D) Correlations between the mean self-reported emotional strength of the faces and the decreased (left, the negative condition) and increased (right, the positive condition) BOLD signal in V1 (compared with the neutral condition) across individual subjects. (E) BOLD signal amplitudes in V1 evoked by nearby gratings (left) and by far gratings (right). (F and G) Correlations between the V1 BOLD signal changes modulated by emotional valence and gain modulation of attentional selection in psychophysical performance. (H and J) The whole-brain search for the pulvinar and DLPFC, with both showing opposite modulations for negative and positive emotions, and their BOLD signal amplitudes. (I and K) Correlations between the V1 BOLD signal changes modulated by emotional valence and that in the pulvinar and DLPFC across individual subjects. Error bars denote 1 SEM calculated across subjects. The data are in the Supporting Information (see S2 Data and S10 Data).

Regions of interest (ROIs) in V1 were defined as the cortical regions responding significantly to the target face, nearby gratings, and far gratings (Fig 3B). We focused our analysis on V1 because activated areas in extrastriate cortex that corresponded to these three different stimuli showed a great deal of overlap. BOLD signals were extracted from these ROIs and then averaged according to emotional valence. For each stimulus block, the 2 s preceding the block served as a baseline, and the mean BOLD signal from 5 s to 16 s after stimulus onset was used as a measure of the response amplitude. The BOLD amplitudes in V1 evoked by the target face and flanking gratings (nearby + far) are shown in Fig 4B and 4C, respectively, and were submitted to a repeated-measures ANOVA with emotional valence as a within-subjects factor. For the target face, the main effect of emotional valence was not significant (F_2, 28_ = 2.416, *p* = 0.112). For the flanking gratings, however, the main effect of emotional valence was significant (F_2, 28_ = 16.582, *p* = 0.001); post hoc paired *t* tests revealed that V1 response during the neutral condition was significantly lower than that during the positive condition (t_14_ = −4.165, *p* = 0.003) but significantly higher than that during the negative condition (t_14_ = 3.806, *p* = 0.006). We further evaluated the role of emotional valence in the modulation of V1 responses to flanking gratings and calculated the correlation coefficients between the self-reported emotional strength of the faces and fMRI measures across individual subjects. Compared to the neutral condition, the decreased BOLD signal in the negative condition and the increased BOLD signal in the positive condition correlated significantly with the self-reported emotional strength of negative faces (r = −0.746, *p* = 0.001, Fig 4D, left) and positive faces (r = 0.633, *p* = 0.011, Fig 4D, right), respectively. Moreover, these decreased and increased BOLD signals also correlated significantly with the response-gain (Fig 4F, left) and contrast-gain (Fig 4G, right) changes, respectively, in the psychophysical experiment.

Our results thus indicated that negative emotion decreased and positive emotion increased the encoding of flanking gratings, as indexed by the BOLD signal changes in V1 evoked by four gratings (nearby + far). However, at least three potential mechanisms could explain the same result: (1) emotional valence modulates the scope of perceptual encoding, with negative emotion narrowing and positive emotion broadening the attention field (S4A Fig); (2) emotional valence modulates the brain state (e.g., arousal), with negative emotion decreasing and positive emotion increasing the V1 signal (S4B Fig); or (3) a combination of hypotheses 1 and 2, with negative emotion narrowing the attention field and decreasing the V1 signal and positive emotion broadening the attention field and increasing the V1 signal (S4C Fig). Accordingly, for each emotional condition, we analyzed the BOLD amplitudes in V1 evoked by nearby gratings and far gratings separately. We hypothesized that these different mechanisms would show different patterns in V1 responses to nearby gratings and far gratings (S4 Fig). The BOLD amplitudes in V1 evoked by nearby gratings and far gratings are shown in Fig 4E (left and right, respectively) and were submitted to a repeated-measures ANOVA with emotional valence (negative, neutral, and positive) and grating distance (nearby and far) as within-subjects factors. The main effect of emotional valence (F_2, 28_ = 17.227, *p* = 0.001), the main effect of the grating distance (F_1, 14_ = 8.140, *p* = 0.013), and the interaction between these two factors (F_2, 28_ = 8.887, *p* = 0.003) were all significant. Thus, these data were submitted to a further simple effect analysis. For the nearby gratings, the main effect of emotional valence was significant (F_2, 28_ = 13.487, *p* = 0.002); post hoc paired *t* tests revealed that there was no significant difference between neutral and positive conditions (t_14_ = −1.866, *p* = 0.250), and both were significantly higher than the negative condition (neutral versus negative: t_14_ = 5.211, *p* < 0.001; positive versus negative: t_14_ = 3.672, *p* = 0.008). For the far gratings, the main effect of emotional valence was also significant (F_2, 28_ = 18.989, *p* < 0.001); post hoc paired *t* tests revealed that there was no significant difference between negative and neutral conditions (t_14_ = −1.900, *p* = 0.235), and both were significantly lower than the positive condition (negative versus positive: t_14_ = −4.322, *p* = 0.002; neutral versus positive: t_14_ = −6.426, *p* < 0.001). For both the negative and neutral conditions, the nearby gratings were significantly higher than the far gratings (negative: t_14_ = 2.849, *p* = 0.013; neutral: t_14_ = 3.366, *p* = 0.005), but significant for the positive condition (t_14_ = 2.160, *p* = 0.049). These findings are consistent with the first hypothesis that emotional valence modulates the scope of perceptual encoding in V1 by narrowing and broadening the attention field.

To examine potential cortical or subcortical area(s) that showed a consistent pattern of activation with that in V1, where negative and positive emotions modulated its responses to flanking gratings in opposite ways (Fig 4C), we performed a group analysis and did a whole-brain search for cortical and subcortical area(s) that showed opposite modulations of flanking gratings for negative and positive emotions, relative to the neutral condition. The results showed that only early visual cortical areas, the pulvinar thalamic nucleus, and DLPFC demonstrated this effect. The BOLD amplitudes in the pulvinar and DLPFC for the three types of emotional valence are shown in Fig 4H and 4J, respectively, and were submitted to a repeated-measures ANOVA with emotional valence as a within-subjects factor. The main effect in both the pulvinar (F_2, 28_ = 9.092, *p* = 0.001) and DLPFC (F_2, 28_ = 23.081, *p* < 0.001) was significant; post hoc paired *t* tests revealed that, for the pulvinar, the negative condition was significantly higher than that during the positive condition (t_14_ = 3.801, *p* = 0.006), but no significant difference was found between the neutral and negative conditions or between the neutral and positive conditions (all *p* > 0.05). For the DLPFC, however, the neutral condition was significantly lower than that during the negative condition (t_14_ = −5.336, *p* < 0.001) but significantly higher than that during the positive condition (t_14_ = 3.779, *p* = 0.006). Furthermore, we found that V1 responses to flanking gratings were significantly correlated with DLPFC responses (Fig 4K), but not with pulvinar responses (Fig 4I). Compared to the neutral condition, V1’s decreased BOLD signal in the negative condition and increased BOLD signal in the positive condition correlated significantly with DLPFC`s increased BOLD signal in the negative condition (r = −0.631, *p* = 0.012) and decreased BOLD signal in the positive condition (r = −0.704, *p* = 0.003), respectively. Taken together, these findings suggest that the modulation of the attention field size in V1 by emotional valence may be derived by feedback from DLPFC.

Additionally, to further exclude the possibility that emotional valence modulation of the attention field size in V1 could be derived from feedback from other attention-specific (i.e., the frontal eye field [FEF] and the posterior parietal cortex [PPC]) or emotion-specific (i.e., the amygdala and medial orbitofrontal cortex [mOFC]) cortical areas, we performed a supplemental analysis and found that the BOLD responses in both the amygdala and mOFC, but not in either FEF or PPC, were significantly modulated by emotional valence. For the amygdala, as well as mOFC, both the negative and positive conditions were significantly higher than the neutral condition; however, no significant difference was found between these two conditions (S5 Fig), showing an inconsistent pattern of activation with that in V1, where the negative condition was significantly lower than the positive condition.

### Effective Connectivity Analyses

**Fig 5:**
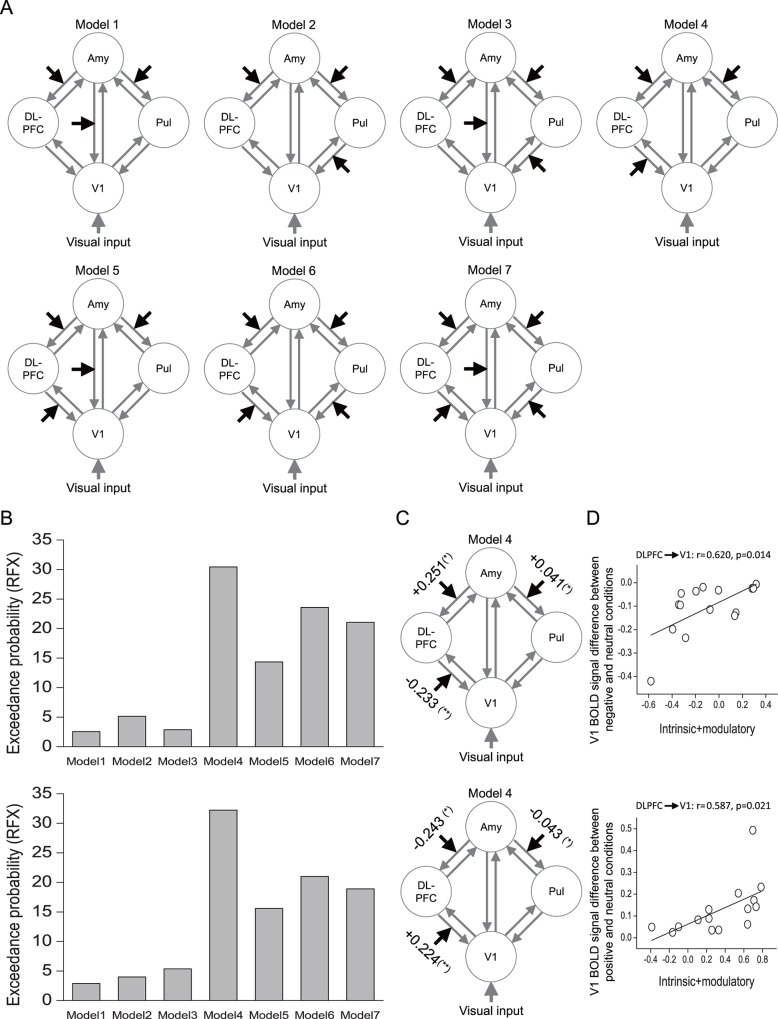
DCM of connectivities among the amygdala, DLPFC, the pulvinar, and V1. (A) Seven different models for modeling the modulatory effect of negative and positive emotions. Amy, amygdala; Pul, pulvinar. (B) Exceedance probabilities for the seven models with negative (up) and positive (down) emotions as the modulatory input. (C) The strength of the modulatory connections for negative (up) and positive (down) emotions and its significance levels (*p < 0.05 and **p < 0.01, respectively). (D) Correlations between the V1 BOLD signal changes of emotional valence and effective connection strengths across individual subjects. The data are in the Supporting Information (see S3 Data).

To directly confirm whether emotional valence modulated the attention field size in V1 through the modulation of feedback from DLPFC, we used dynamic causal modeling (DCM) to examine functional changes in directional connectivity among the amygdala, DLPFC, the pulvinar, and V1 related to negative and positive emotions. The pulvinar was selected in the models since it showed a consistent pattern of activation with DLPFC (Fig 4H), while the amygdala was selected in the models since it is well known as a critical brain area for emotion processing [24], showing significantly greater responses to emotional faces than neutral faces (S5 Fig). Given the extrinsic visual input into V1, we defined seven different models with modulatory inputs (either the negative emotion or positive emotion, Fig 5A). The modulatory inputs could modulate the feedback from the amygdala (Model 1), from the pulvinar (Model 2), from both the amygdala and pulvinar (Model 3), from DLPFC (Model 4), from both the amygdala and DLPFC (Model 5), from both DLPFC and the pulvinar (Model 6), and from all three areas (Model 7) to V1. We examined these seven models for modeling the modulatory effect by negative and positive emotions and fit each of these seven models for each subject.

For negative emotion, we computed the exceedance probability of each model [39]. The result showed that Models 1 through 7 had exceedance probabilities of 2.55%, 5.14%, 2.88%, 30.45%, 14.35%, 23.57%, and 21.05%, respectively, suggesting that Model 4 was the best one to explain the modulatory effect by negative emotion (Fig 5B, up). The negative emotion significantly increased the feedback connectivity from the amygdala to both DLPFC (t_14_ = 2.906, *p* = 0.011) and the pulvinar (t_14_ = 2.213, *p* = 0.044) but decreased the feedback connectivity from DLPFC to V1 (t_14_ = −3.792, *p* = 0.002) (Fig 5C, up). For positive emotion, the exceedance probabilities of Model 1 to Model 7 were 2.89%, 3.98%, 5.37%, 32.24%, 15.60%, 21.02%, and 18.91%, respectively, suggesting that the modulatory effect by positive emotion was also best explained by Model 4 (Fig 5B, down). However, the positive emotion significantly decreased the feedback connectivity from the amygdala to both DLPFC (t_14_ = −2.743, *p* = 0.016) and the pulvinar (t_14_ = −2.573, *p* = 0.022) but increased the feedback connectivity from DLPFC to V1 (t_14_ = 3.923, *p* = 0.002) (Fig 5C, down). Furthermore, we calculated the correlation coefficients between V1 responses and the effective connection strengths (the sum of the intrinsic and modulatory connectivities) from DLPFC to V1 across individual subjects. Compared to the neutral condition, the decreased V1 BOLD signal in the negative condition (r = 0.620, *p* = 0.014) and the increased V1 BOLD signal in the positive condition (r = 0.587, *p* = 0.021) correlated significantly with feedback connectivity from DLPFC to V1 (Fig 5D). Additionally, a supplemental DCM analysis with mOFC, instead of the amygdala, showed significantly greater responses to emotional faces than neutral faces, confirming these results (S6 Fig). Together, these results further support the idea that emotional valence-dependent modulations of the attention field size in V1 may be derived by feedback from DLPFC.

## Discussion

This study examined whether emotional attention shapes perception via a normalization framework. The normalization model of attention proposes that attention can affect performance by response- or contrast-gain changes, depending on the stimulus size and the attention field size [19]. Previous studies have suggested that negative emotion could narrow and positive emotion could broaden the scope of perceptual encoding [29,31,32], which offers a unique opportunity to change the size of the attention field relative to the stimulus size. Here, we measured the gain pattern of CRFs on the spatial cueing effect derived from negative and positive faces. We found a change in the spatial cueing effect consistent with a change in response gain for negative faces and in contrast gain for positive faces. The fMRI experiment confirmed that emotional valence modulated the attention field in V1; negative faces decreased and positive faces increased V1 responses to flanking gratings. Importantly, across subjects, the self-reported emotional strength of negative and positive faces correlated, respectively, both with response- and contrast-gain changes and with V1 decreased and increased responses to flanking gratings. Furthermore, effective connectivity analysis showed that the V1 attention field size controlled by emotional valence was best explained by increased and decreased feedback from DLPFC to V1.

Our data provide, to our knowledge, the first neural evidence that emotional attention interacts with normalization processes depending on emotional valence. Our behavioral data can be interpreted by a hypothesis that behavioral performance is limited by the neuronal activity with an additive, independent, and identically distributed noise, and the decision-making process with a maximum-likelihood decision rule [40]. Performance accuracy *d'*, used in both Herrmann et al. [17] and our studies, is proportional to the signal-to-noise ratio of the underlying neuronal responses. Thus, it can reflect in parallel any change in neuronal CRFs in our study. Indeed, we found that a change in the cueing effect (Fig 2A) was consonant with a change in response gain of CRF (Fig 1B) for negative faces and a change in contrast gain of CRF (Fig 1C) for positive faces. These emotional valence-dependent gain modulations of attentional selection not only are consistent with existing psychophysical [30,32,41] and brain imaging [31,42] studies suggesting that negative emotion narrows and positive emotion broadens the scope of perceptual encoding, but also support and extend the normalization model of attention [19]. This model proposes that, in the absence of attention (e.g., in the incongruent cue condition), two factors determine the firing rate of a visually responsive neuron. One is the stimulus drive (excitatory component) determined by the contrast of the stimulus placed in the receptive field of a neuron. The other is the suppressive drive (inhibitory component) determined by the summed activity of other neighboring neurons, which serves to normalize the overall spike rate of the given neuron via mutual inhibition [43,44]. Attention (e.g., in the congruent cue condition) modulates the pattern of neural activity by altering the balance between these excitatory and inhibitory components, depending on the relative sizes of the attention field to the stimulus size, and thereby exhibiting response-gain changes, contrast-gain changes, and various combinations of the two. In our study, given the fixed size of the target stimuli in the spatial cueing task, the narrowed attention field by negative emotion led to response-gain changes because attentional gain enhanced the entire stimulus drive but enhanced only the center of the suppressive drive. Conversely, the broadened attention field by positive emotion led to contrast-gain changes because attentional gain was applied equally to the stimulus and suppressive drives.

The fMRI data confirmed these emotional valence-dependent changes of the attention field; negative emotion narrowed and positive emotion broadened the attention field in V1. Importantly, this result cannot be explained by brain state changes or by the combination of brain state and attention field changes (S4 Fig). Moreover, the result cannot be explained by a number of other factors, such as low-level features, task difficulty, target face processing, or eye movement. First, the size and contrast of the flanking gratings were identical on any given trial, and the phase and orientation were random across trials, suggesting no physical difference of the gratings among the different emotional conditions. Second, during scanning, the flanking gratings were never task relevant for the subjects, who performed a gender discrimination task on the faces. There was no significant difference in subject performance among the three types of emotional valence of the faces, suggesting no difference in task difficulty. Third, the finding of no significant activation difference in V1 for the emotional faces excluded the possibility of a trade-off between attention to the target face and flanking gratings (Fig 4B). Finally, the eye movement data showed that the subjects’ eye movements were small and their eye position distributions were statistically indistinguishable for the three types of emotional valence (S3 Fig). Although our eye movement data were recorded in a psychophysics lab (outside the scanner), it should be noted that the recordings were made when subjects performed the same task as the one in the fMRI experiments. Differences in eye movements for the three types of emotional valence may be a potential confound, but it is highly unlikely since our recordings outside the scanner did not detect any such differences.

One should note that emotional valence-dependent modulations of attention fields in our study were indexed by the decreased and increased V1 responses to flanking gratings, which were irrelevant and presumably ignored while subjects attended to the target face. Thus, how does emotional valence differentially modulate V1 responses to these distractors? Previous neurophysiological and brain neuroimaging studies have implicated prefrontal areas in the filtering of distractors [45–50], and our findings are consistent with such an influence. Our findings suggest that distractor suppression by emotional valence in V1 could be associated with feedback from DLPFC. First, DLPFC responses were significantly modulated by emotional valence and showed a pattern of activation consistent with that in V1, where negative and positive emotions modulated its responses to task-irrelevant distractors in opposite ways. This consistent pattern of activation between V1 and DLPFC was also confirmed by a group analysis and a whole-brain search for cortical and subcortical area(s) that showed opposite responses for negative and positive emotions (Fig 4J). Second, V1 responses to flanking distractors were significantly predicted by DLPFC responses (Fig 4K). Finally, the DCM analysis indicated that negative emotion increased and positive emotion decreased suppression from DLPFC to V1, and these suppression effects significantly predicted the V1 responses to flanking distractors (Fig 5C and 5D).

Our study succeeded in linking emotional valence-dependent feedback from the DLPFC to V1 directly with distractor suppression. Based on our fMRI findings, in conjunction with existing neurophysiological [51], behavioral [30,32], and neuroimaging [42,52] data, we speculate that emotional valence-dependent distractor suppression is derived from DLPFC influences on the scope of inhibitory control. Inhibitory control is thought to limit the amount of information entering the focus of attention [53], which, in turn, affects the scope of attentional selection, and DLPFC is thought to play a very important role in this function [48]. We speculate that, in our study, when the emotional faces were negative, both feedback from the amygdala to DLPFC and the BOLD signal in DLPFC increased and thus decreased effective connectivity (i.e., increased suppression) from DLPFC to V1, as revealed by the DCM analysis, which then would increase the inhibitory control, in other words, increase the inhibition of ignored distractors, resulting in a narrowed focus of attention and reduced processing of the flanking gratings. Conversely, when the emotional faces were positive, both feedback from the amygdala to DLPFC and the BOLD signal in DLPFC decreased and thus increased effective connectivity (i.e., decreased suppression) from DLPFC to V1, which would decrease the inhibition of ignored distractors, resulting in a broadened scope of attention and flanking gratings that were more fully processed. It should be noted that our speculation only provides a possible mechanism for emotional valence-dependent attention field in V1, which should be tested with neurophysiological techniques in the future.

Our results also indicate that the pulvinar may be involved in emotional valence-dependent modulations of distractor suppression in V1, consistent with previous lesion [54], neurophysiological [55], and brain neuroimaging [56] studies, implicating the pulvinar’s involvement in the filtering of unwanted information. However, it is important to note that the ROIs in the pulvinar defined in our study were across dorsal and ventral parts. The dorsal pulvinar predominantly projects to areas within the frontoparietal network and superior anterior temporal cortex [57]; the ventral pulvinar, conversely, exhibits reciprocal connections with successive occipitotemporal cortical areas along the ventral processing stream [58,59]. Thus, further work is needed to use high-spatial resolution fMRI or neurophysiological techniques to parse the relative contributions of the dorsal and ventral pulvinar to emotional valence-dependent modulations of distractor suppression.

One should note that our results cannot be explained by a number of other factors, including poststimulus modulation by the response cue, greater attention directed to negative faces, or an effect of emotional faces on decisional rather than perceptual processing of the target. First, although previous studies have suggested that the poststimulus cue (for example, the response cue in our study) can influence not only subjects’ nonperceptual decision [60] but also the perception of stimuli presented before it [15,61,62], the response cue in our study was totally randomized and uninformative about the target; we thus believe that our psychophysical results cannot be explained by the response cue. Second, although previous studies have found that negative faces tend to attract more attention and show a greater response than positive faces [25,26], this effect was not obtained in our study; no significant difference in response to negative and positive faces was found in V1 (Fig 4B), the amygdala, or mOFC (S5 Fig), thus eliminating the specific impact of negative faces as a factor affecting our fMRI results. Finally, if the emotional faces (negative versus positive) affected subjects’ decisional rather than their perceptual processing of the target stimuli, then these two conditions should have produced different responses in the orbitofrontal cortex (OFC), an area critically involved in decision making [63,64]; however, no significant difference between these two conditions was found in OFC (S5 Fig), indicating that the observed difference between the negative and positive conditions was most likely caused by perceptual rather than decision-making processes. In addition, our study used the normalization model to predict and explain psychophysical performance only. Our fMRI experiment did not measure the BOLD response for different contrast levels but instead examined whether negative emotion narrowed and positive emotion broadened subjects’ attention fields. The design of our fMRI study took into account the results of several papers reporting that the attentional effect on the BOLD response is constant across different contrast levels, showing a baseline increase/additive shift rather than either a response gain or contrast gain [12,14,15]. In those studies, the attention field was manipulated by focused (narrowed attention field) and distributed (broadened attention field) cues. These two cues, however, either enhanced the entire contrast range or produced a combination of both response-gain and contrast-gain changes, indicating inconsistent predictions of the normalization model [19]. Previous studies have suggested that their results may be because BOLD signals integrate the activity across neurons showing different attention modulatory effects, which would result in various combinations of both response and contrast gains [15,16]. Moreover, attention-triggered BOLD signals can be driven by both bottom-up stimuli and top-down goals [14,65]; hence, the increased BOLD signals to the attended low and mid-contrast stimuli may be mainly driven by the top-down modulation rather than the bottom-up stimuli.

In sum, our study provides strong evidence that gain modulation of emotional attention depends on emotional valence. Negative emotion and positive emotion modulate the attention field in V1 in opposite ways, maybe depending on the increased or decreased feedback from DLPFC, thereby changing the suppression of distractors [53]. The prominent role of the prefrontal cortex in distractor suppression evident here is consistent with recent neurophysiological findings that have begun to address how prefrontal areas directly influence sensory representations to filter out distractors [49,51,66]. Identifying DLPFC as a potential neural substrate of emotional valence-dependent normalization processing of attention gives insight into how the interaction between emotion and attention shapes our experience of the world.

## Materials and Methods

### Subjects

A total of 23 human subjects (8 males, 21–41 y old) participated in the study. All 23 participated in the psychophysical experiment, and 15 (8 males, 21–41 y old) of them participated in the fMRI experiment. All subjects were naїve to the purpose of the study. They reported normal or corrected-to-normal vision and had no known neurological, psychiatric, or visual disorders. They gave written informed consent in accordance with protocols approved by the National Institute of Mental Health (NIMH) Institutional Review Board (93-M-0170).

### Stimuli

Forty angry, forty happy, and forty neutral faces were chosen from the NimStim Set of Facial Expressions (http://www.macbrain.org/resources.htm) [36]. All faces were masked to exclude ears, neck, hair, and hairline and were scaled to the same size (diameter: 2.2°) (Fig 1A). In the psychophysical experiment, a pair of faces were centered in the left and right hemifields at 4.65° eccentricity, one of which was an emotional face. Target gratings (spatial frequency: 4.0 cycles/°; diameter: 2.2°; phase: random) were presented at five possible contrasts: 0.03, 0.08, 0.20, 0.45, and 0.75. In the fMRI experiment, a single face (diameter: 2.2°) was centered in either the left or right hemifield at 4.65° eccentricity and was flanked by four gratings (diameter: 1.4°; spatial frequency: 4.0 cycles/°; contrast: 0.20; phase: random; orientation: randomly chosen from 0° to 180°). The center-to-center distance between the face and nearby gratings and between the face and far gratings was 2.54° and 4.52°, respectively (Fig 3A and 3B).

### Psychophysical Experiments

Visual stimuli were displayed on a BENQ LCD monitor (model: XL2420Z; refresh rate: 60 Hz; resolution: 1,920 × 1,080; size: 24 in) at a viewing distance of 57 cm. The subjects’ head position was stabilized using a chin rest. A white fixation (diameter: 0.1°) cross was always present at the center of the monitor.

Each trial began with central fixation. A pair of faces (one emotional) were presented in the left and right hemifields for 150 ms, followed by a 50 ms fixation interval. The emotional face served as a cue to attract covert spatial attention. Then, a pair of gratings (with identical contrasts) were presented for 33 ms in the left and right hemifields at 4.65° eccentricity, one of which was the target. Subjects were asked to press one of two buttons to indicate the orientation of the grating (leftward or rightward tilted) and received auditory feedback if their response was incorrect. Target location was indicated by a peripheral 100 ms response cue (0.5° white line) above one of the grating locations, but not at the grating location to avoid masking. A congruent cue was defined as a match between the emotional face location and response cue location (half the trials); an incongruent cue was defined as a mismatch (half the trials). Participants were explicitly told that the emotional faces were randomized and uninformative about the target location (Fig 1A). The experiment consisted of two sessions (negative emotion and positive emotion of the faces), with the two sessions occurring on different days; the order of the two sessions was counterbalanced across subjects. Each session consisted of 30 blocks; each block had 80 trials, from randomly interleaving 16 trials from each of the five contrasts. Contrast varied from trial to trial in randomly shuffled order, and stimuli were presented briefly (i.e., 33 ms) to avoid any possible dependence of attentional state on stimulus contrast. The attentional effect for each grating contrast was quantified as the difference between the performance accuracy (*d'*) in the congruent and incongruent cue conditions. After each session, subjects were asked to rate (on a seven-point Likert scale) their self-perception of the emotional strength of each emotional face. For each subject, the self-reported emotional strength of positive and negative emotion was the mean rating for 40 positive and 40 negative faces, respectively.

### Psychophysical Data Analysis

To quantitatively examine the pattern of gain (either response or contrast gain) separately for positive emotion and negative emotion, for each subject, performance—i.e., *d'* = z (hit rate)–z (false alarm rate)—was assessed across experimental blocks for each contrast and each trial condition (congruent and incongruent). A rightward response to a rightward stimulus tilt was (arbitrarily) considered to be a hit, and a rightward response to a leftward stimulus was considered to be a false alarm. For each subject, the mean *d'* contrast response functions (CRFs) obtained for congruent and incongruent trials were fit with the standard Naka–Rushton equation [37]:

$$d\prime \;(c)=d\prime_{max}\;(c^{n}\;/\;[c^{n}+c_{50}{}^{n}]),$$

where *d'* is performance as a function of contrast (*c*), *d'*_*max*_ determines the asymptotic performance at high contrasts, *c*_50_ is the contrast corresponding to half the asymptotic performance, and *n* is an exponent that determines the slope of the CRFs. In this analysis, *n* was fixed at 2 [17,38].

### fMRI Experiments

Using a block design, the experiment consisted of six functional runs. Each run consisted of 12 stimulus blocks of 16 s, interleaved with 12 blank intervals of 16 s. There were 6 different stimulus blocks: 2 (visual field: left/right) × 3 (emotional valence: negative/neutral/positive). Each stimulus block was randomly repeated two times in each run, and consisted of 8 trials; on each trial, a face flanked by four gratings was presented for 0.3 s, followed by a 1.7-s fixation interval, and subjects were asked to discriminate the gender of the face (male or female) while maintaining central fixation throughout the trial (Fig 3C).

The V1 boundary was defined by a standard phase-encoded method developed by Sereno et al. [67] and Engel et al. [68], in which subjects viewed rotating wedge and expanding ring stimuli that created traveling waves of neural activity in visual cortex. A block-design scan was used to localize the ROIs in V1 corresponding to the target face, nearby gratings, and far gratings (Fig 3B). The localizer scan consisted of 12 stimulus blocks of 12 s, interleaved with 12 blank intervals of 12 s. In a stimulus block, subjects passively viewed 8-Hz flickering patches. Each block type was repeated four times in the run, which lasted 288 s.

### MRI Data Acquisition

MRI data were collected using a 3T Siemens Trio scanner with a 32-channel phase-array coil. In the scanner, the stimuli were back-projected via a video projector (refresh rate: 60 Hz; spatial resolution: 1,280 × 800) onto a translucent screen placed inside the scanner bore. Subjects viewed the stimuli through a mirror located above their eyes. The viewing distance was 115 cm. BOLD signals were measured with an echo-planar imaging sequence (TR: 2,000 ms; TE: 30 ms; FOV: 192 × 192 mm^2^; matrix: 64 × 64; flip angle: 90°; slice thickness: 3 mm; gap: 0 mm; number of slices: 34; slice orientation: axial). The bottom slice was positioned at the bottom of the temporal lobes. A 3D MPRAGE structural dataset (resolution: 1 ×1 × 1 mm^3^; TR: 2,600 ms; TE: 30 ms; FOV: 256 × 224 mm^2^; flip angle: 8°; number of slices: 176; slice orientation: sagittal) was collected in the same session before the functional scans. Subjects underwent two sessions, one for retinotopic mapping and the other for the main experiment.

### MRI Data Analysis

The anatomical volume for each subject in the retinotopic mapping session was transformed into a brain space that was common for all subjects [69] and then inflated using BrainVoyager QX. Functional volumes in both sessions for each subject were preprocessed, including 3-D motion correction, linear trend removal, and high-pass (0.015 Hz) filtering using BrainVoyager QX [70]. Head motion within any fMRI session was <2 mm for all subjects. The images were then aligned to the anatomical volume from the retinotopic mapping session and transformed into Talairach space [69]. The first 8 s of BOLD signals were discarded to minimize transient magnetic saturation effects. A general linear model (GLM) procedure was used to determine the V1’s boundary and ROI analysis. V1 boundaries were delineated by a standard retinotopic mapping method [67,68]. The ROIs within V1 were defined as regions that responded more strongly to the flickering patches than to the blank screen (*p* < 10^−3^, uncorrected).

### DCM

To directly confirm whether emotional valence modulated the attention field size in V1 through the modulation of feedback from DLPFC, we applied DCM analysis in SPM10 to our fMRI data [39]. For each subject and each hemisphere, using BrainVoyager QX, the amygdala and V1 (including dorsal and ventral parts) voxels were identified as those activated by the emotional block and the flanking gratings at a significance level of *p* < 0.005, respectively; both the pulvinar and DLPFC voxels were identified as those activated by the stimulus block at a significance level of *p* < 0.005. The mean Talairach coordinates of these voxels and the standard errors across subjects in the amygdala, dorsal V1, ventral V1, the pulvinar, and DLPFC were [−22 ± 1.4, −7 ± 1.0, −13 ± 1.1], [−7 ± 0.8, −93 ± 1.0, −12 ± 1.3], [−3 ± 1.1, −84 ± 1.1, −16 ± 1.2], [−18 ± 1.9, −27 ± 1.2, 7 ± 0.9], and [−44 ± 1.6, 25 ± 1.7, 29 ± 2.7] for the left hemisphere and [25 ± 1.6, −9 ± 1.0, −15 ± 1.0], [7 ± 1.1, −94 ± 0.6, −8 ± 2.2], [3 ± 0.8, −83 ± 1.1, −14 ± 1.7], [17 ± 1.7, −29 ± 1.0, 7 ± 0.9], and [45 ± 1.7, 21 ± 3.1, 31 ± 2.3] for the right hemisphere, respectively. For each subject and each hemisphere, these Talairach coordinates were converted to Montreal Neurological Institute (MNI) coordinates using the tal2mni conversion utility (http://imaging.mrc-cbu.cam.ac.uk/downloads/MNI2tal/tal2mni.m). In SPM, for each of these areas, we extracted voxels within a 4-mm sphere centered on the most significant voxel and used their time series for the DCM analysis. The estimated DCM parameters were later averaged across dorsal and ventral V1 and the two hemispheres using the Bayesian model averaging method [39].

DCMs have three sets of parameters: (1) extrinsic input into one or more regions; (2) intrinsic connectivities among the modeled regions; and (3) bilinear parameters encoding the modulations of the specified intrinsic connections by experimental manipulations [39,71,72]. The third set of parameters is used to quantify modulatory effects, which reflect increases or decreases in connectivity between two regions given some experimental manipulation, compared with the intrinsic connections between the same regions that capture connectivity in the absence of experimental manipulation. FMRI data were modeled using GLM, with regressors for negative, neutral, and positive emotions, as well as a fourth condition comprising all visual input. The fourth condition was added specifically for the DCM analysis to be used as a direct visual input. Given the extrinsic visual input into V1, we defined seven different models with modulatory inputs (either the negative emotion or positive emotion, Fig 5A). The modulatory inputs could modulate feedback from the amygdala (Model 1), from the pulvinar (Model 2), from both the amygdala and pulvinar (Model 3), from DLPFC (Model 4), from both the amygdala and DLPFC (Model 5), from both DLPFC and the pulvinar (Model 6), and from all three areas (Model 7) to V1. We examined these seven models for modeling the modulatory effect by negative and positive emotions. We fit each of these seven models for each subject. Using a hierarchical Bayesian approach [73], we compared the seven models by computing the exceedance probability of each model, i.e., the probability to which a given model is more likely than any other included model to have generated data from a randomly selected subject [39,71,72]. In the best model (Model 4), we examined the modulatory effects by negative and positive emotions.

### Eye Movement Recordings

Eye movements were recorded with an ASL EyeTrac 6000 (Applied Science Laboratories, Bedford, Massachusetts) in a psychophysics lab (outside the scanner). Its temporal resolution was 60 Hz, and its spatial resolution was 0.25°. Recording was performed when subjects performed the same task as the psychophysical and fMRI experiments. S1 Fig and S3 Fig show that subjects’ eye movements were small and statistically indistinguishable across all conditions.

## Supporting Information

S1 Data Data for Fig 2. (XLSX)

S2 Data Data for Fig 4. (XLSX)

S3 Data Data for Fig 5. (XLSX)

S4 Data Data for S1 Fig. (XLSX)

S5 Data Data for S2 Fig. (XLSX)

S6 Data Data for S3 Fig. (XLSX)

S7 Data Data for S4 Fig. (XLSX)

S8 Data Data for S5 Fig. (XLSX)

S9 Data Data for S6 Fig. (XLSX)

S10 Data The dataset contains the Brain Voyager t-value images (vmp and nifti-1 formats) of the statistical maps of Fig 4 and S5 Fig and the individual fMRI data. (ZIP)

S1 Fig Eye movement data for the psychophysical experiment. Histograms of horizontal (up) and vertical (down) eye positions after removing blinks and artifacts in the congruent (A) and incongruent (B) cues. A congruent cue was defined as a match between the emotional face location and response cue location; an incongruent cue was defined as a mismatch. Eye movements were small, and eye position distributions were very similar between the negative and positive conditions. *T* tests showed that the horizontal and vertical mean eye positions of all the distributions did not deviate significantly from the fixation point (all *p* > 0.05). The data are in the Supporting Information (see S4 Data).
(EPS)

S2 Fig Spatial cueing effects on performance (*d'*) as a function of contrast for the negative first group and the positive first group. Negative first group: subjects finished the negative session (negative faces) in the first day and the positive session (positive faces) in the second day. Positive first group: subjects finished these two sessions in the opposite order. (A) Mean *d'* plotted as psychometric functions of stimulus contrast, session order, and emotional valence for congruent and incongruent trials. (B) Correlations between the mean self-reported emotional strength of the faces and the *d'*_*max*_ differences between congruent and incongruent trials across individual subjects. (C) Correlations between the mean self-reported emotional strength of the faces and the *c*_*50*_ differences between congruent and incongruent trials across individual subjects. (D) *d'*_*max*_ and (E) *c*_*50*_ for trial conditions, session order, and emotional valence. Error bars denote 1 SEM calculated across subjects. Both *d'*_max_ and *c*_*50*_ were submitted to a three-way mixed ANOVA with session order (negative first and positive first) as the between-subjects factor and emotional valence (negative and positive) and trial condition (congruent and incongruent) as the within-subjects factors. For *d'*_max_, the interaction between session order and emotional valence (F_1, 21_ = 0.028, *p* = 0.869), the interaction between session order and trial condition (F_1, 21_ = 0.007, *p* = 0.934), and the interaction among these three factors (F_1, 21_ = 0.010, *p* = 0.919) were all insignificant. Similarly, for *c*_*50*,_ the interaction between session order and emotional valence (F_1, 21_ = 0.194, *p* = 0.664), the interaction between session order and trial condition (F_1, 21_ = 0.073, *p* = 0.790), and the interaction among these three factors (F_1, 21_ = 1.569, *p* = 0.224) were all insignificant. These results suggest that the order of the two sessions did not influence our psychophysical results. The data are in the Supporting Information (see S5 Data).
(EPS)

S3 Fig Eye movement data for the fMRI experiment. Histograms of horizontal (up) and vertical (down) eye positions after removing blinks and artifacts when the stimuli were presented in the left (A) and right (B) visual fields. Their eye movements were small, and eye position distributions were very similar among the negative, neutral, and positive conditions. *T* test analyses showed that the horizontal and vertical mean eye positions of all the distributions did not deviate significantly from the fixation point (all *p* > 0.05). The data are in the Supporting Information (see S6 Data).
(EPS)

S4 Fig Three hypotheses about the modulation of V1 responses to irrelevant flanking gratings by emotional valence. (A) Hypothesis 1: emotional valence modulates the scope of perceptual encoding, with negative emotion narrowing and positive emotion (the faces of a coauthor here are for illustration purposes only; they were not used in the experiments) broadening the attention field (the diameter of disk indicates the size of the attention field). (B) Hypothesis 2: emotional valence modulates the brain state (e.g., arousal), with negative emotion decreasing and positive emotion increasing the V1 signal (the gray level of disk indicates the level of brain signal). (C) Hypothesis 3: a combination of hypotheses 1 and 2, with negative emotion narrowing the attention field and decreasing the V1 signal and positive emotion broadening the attention field and increasing the V1 signal. We predicted that these hypotheses could explain the same result, i.e., that negative emotion decreases and positive emotion increases the encoding of the irrelevant flanking gratings, as indexed by the BOLD signal changes in V1 evoked by the nearby and far gratings (D, E, and F, left). However, when analyzing BOLD signals in V1 evoked by nearby gratings and far gratings separately, these different hypotheses should show different patterns (D, E, and F, right). First, if emotional valence modulates the scope of perceptual encoding (A and D), then for the nearby gratings, V1 should show a lower response in the negative condition than that in the neutral and positive conditions, but no significant difference between the neutral and positive conditions should be found. However, for the far gratings, V1 should show a higher response in the positive condition than that in the negative and neutral conditions, but no significant difference between the negative and neutral conditions should be found. Second, if emotional valence modulates the brain state (B and E), for both the nearby and far gratings, V1 should show the lowest, median, and highest response in the negative, neutral, and positive conditions, respectively. Third, if emotional valence modulates both the scope of perceptual encoding and the brain state (C and F), for the nearby gratings, V1 should show a similar pattern to that in hypothesis 2, with the lowest, median, and highest response in the negative, neutral, and positive conditions, respectively. However, for the far gratings, V1 should show a similar pattern to that in hypothesis 1, with a higher response in the positive condition than that in the negative and neutral conditions, but no significant difference between the negative and neutral conditions should be found. The data are in the Supporting Information (see S7 Data).
(EPS)

S5 Fig BOLD signal for the three types of emotion valence in the ROIs in the amygdala, mOFC, FEF, and PPC. (A and B) Emotion- and attention-specific cortical/subcortical areas (the results of DLPFC were shown in Fig 4). mOFC: medial orbitofrontal cortex; FEF: frontal eye field; PPC: posterior parietal cortex. The BOLD amplitudes in these areas for the three types of emotional valence were submitted to a repeated-measures ANOVA with emotional valence as a within-subjects factor. The main effect in both FEF (F_2, 28_ = 0.053, *p* = 0.902) and PPC (F_2, 28_ = 2.320, *p* = 0.133) was not significant. However, the main effect in both the amygdala (F_2, 28_ = 14.473, *p* = 0.001) and mOFC (F_2, 28_ = 20.693, *p* < 0.001) was significant; post hoc paired *t* tests revealed that, for both the amygdala and mOFC, the neutral condition was significantly lower than both the negative condition (the amygdala: t_14_ = −4.136, *p* = 0.003; mOFC: t_14_ = −3.419, *p* = 0.012) and the positive condition (the amygdala: t_14_ = −3.674, *p* = 0.008; mOFC: t_14_ = −7.139, *p* < 0.001); no significant difference was found between the negative and positive conditions (the amygdala: t_14_ = 2.243, *p* = 0.125; mOFC: t_14_ = −2.487, *p* = 0.078). Error bars denote 1 SEM calculated across subjects. (C) Correlations between the BOLD signal difference between negative and neutral conditions in V1 and in the amygdala, mOFC, FEF, and PPC across individual subjects. (D) Correlations between the BOLD signal difference between positive and neutral conditions in V1 and in the amygdala, mOFC, FEF, and PPC across individual subjects. The data are in the Supporting Information (see S8 Data and S10 Data).
(EPS)

S6 Fig DCM of connectivities among the mOFC, DLPFC, the pulvinar, and V1. (A) Seven different models for modeling the modulatory effect of negative and positive emotions (mOFC was selected in the models since it also showed significantly greater responses to emotional faces than neutral faces, similar to the amygdala; see S5 Fig). mOFC: medial orbitofrontal cortex (Talairach coordinates: [−11 ± 1.4, 56 ± 1.6, 5 ± 1.6] and [10 ± 1.6, 55 ± 1.9, 6 ± 1.6] for the left and right hemispheres, respectively); Pul: pulvinar. (B) For negative emotion, the exceedance probabilities of Models 1 through 7 were 2.33%, 11.87%, 6.29%, 26.78%, 11.98%, 23.88%, and 16.87%, respectively, suggesting that Model 4 was the best one to explain the modulatory effect by negative emotion (up); for positive emotion, the exceedance probabilities of Models 1 through 7 were 5.60%, 10.72%, 8.79%, 24.21%, 13.10%, 21.09%, and 16.49%, respectively, suggesting that the modulatory effect by positive emotion was also best explained by Model 4 (down). (C) The strength of the modulatory connections for negative (up) and positive (down) emotions, and its significance levels (*p < 0.05 and **p < 0.01, respectively). The negative emotion significantly increased the feedback connectivity from mOFC to both DLPFC (t_14_ = 3.069, *p* = 0.008) and the pulvinar (t_14_ = 2.793, *p* = 0.014) but decreased the feedback connectivity from DLPFC to V1 (t_14_ = −3.295, *p* = 0.005); the positive emotion significantly decreased the feedback connectivity from mOFC to both DLPFC (t_14_ = −2.621, *p* = 0.020) and the pulvinar (t_14_ = −2.880, *p* = 0.012) but increased the feedback connectivity from DLPFC to V1 (t_14_ = 3.688, *p* = 0.002). (D) Correlations between the V1 BOLD signal changes of emotional valence and effective connection strengths across individual subjects. The data are in the Supporting Information (see S9 Data).
(EPS)

## Abbreviations

BOLD: blood oxygenation-level-dependent
CRF: contrast-response function
DCM: dynamic causal modeling
DLPFC: dorsolateral prefrontal cortex
FEF: frontal eye field
fMRI: functional magnetic resonance imaging
MNI: Montreal Neurological Institute
mOFC: medial orbitofrontal cortex
PPC: posterior parietal cortex
ROI: region of interest
SEM: standard error of the mean
V1: primary visual cortex
